# Nitrate of Silver in Diseases of the Eye

**Published:** 1844-07

**Authors:** 


					﻿Nitrate of Silver in Diseases of the Eye.— 1. Nitrate of sil-
ver is the best topical application that can be employed in a
great number of acute and chronic affections of the eye.
2.	In the various forms of blepharitis it should be used as
an ointment.
3.	In inflammations of the eyelids it is most advantageous-
ly applied in the solid form.
4.	In slight inflammations of the conjunctiva a solution of
from five to fifteen centigrammes* of nitrate of silver to thir-
ty grammes of water are generally sufficient.
* The gramme is nearly 19 grains;—centigramme one.fifth of a grain.
5.	In purulent conjunctivitis the dose may be increased
from two grammes to thirty grammes of water.
6.	The use of the solid nitrate of silver may also be bene-
ficial, but is hazardous.
7.	It is always very advantageous, in the treatment of con-
junctivitis to alternately increase and diminish the dose of
nitrate of silver.—Med. Press.
				

